# Genetic dissection of nitrogen induced changes in the shoot and root biomass of spinach

**DOI:** 10.1038/s41598-022-18134-7

**Published:** 2022-08-12

**Authors:** Vijay Joshi, Ainong Shi, Amit Kumar Mishra, Haramrit Gill, James DiPiazza

**Affiliations:** 1 Texas A&M AgriLife Research and Extension Center, Uvalde, TX 78801 USA; 2 Department of Horticulture, University of Arkansas, Fayetteville, AR 72701 USA; 3 Department of Horticultural Sciences, Texas A&M University, College Station, TX 77843 USA; 4 Present Address: Department of Botany, School of Life Sciences, Mizoram University, Aizawl, Mizoram 796004 India

**Keywords:** Plant sciences, Plant breeding

## Abstract

Efficient partitioning of above and below-ground biomass in response to nitrogen (N) is critical to the productivity of plants under sub-optimal conditions. It is particularly essential in vegetable crops like spinach with shallow root systems, a short growth cycle, and poor nitrogen use efficiency. In this study, we conducted a genome-wide association study (GWAS) to explore N-induced changes using spinach accessions with diverse genetic backgrounds. We evaluated phenotypic variations as percent changes in the shoot and root biomass in response to N using 201 spinach accessions grown in randomized complete blocks design in a soilless media under a controlled environment. A GWAS was performed for the percent changes in the shoot and root biomass in response to N in the 201 spinach accessions using 60,940 whole-genome resequencing generated SNPs. Three SNP markers, chr4_28292655, chr6_1531056, and chr6_37966006 on chromosomes 4 and 6, were significantly associated with %change in root weight, and two SNP markers, chr2_18480277 and chr4_47598760 on chromosomes 2 and 4, were significantly associated with % change shoot weight. The outcome of this study established a foundation for genetic studies needed to improve the partitioning of total biomass and provided a resource to identify molecular markers to enhance N uptake via marker-assisted selection or genomic selection in spinach breeding programs.

## Introduction

N is critical to a plant's growth and development as an essential macronutrient. To meet the food demand of the growing human population, the utilization of N fertilizers has become inevitable^1^. However, excessive N inputs through fertilizers have led to N losses to the environment via leaching or gaseous emissions, resulting in adverse impacts on the climate, environment, and human health. Enhancing the NUE of crops has become an apparent strategy to promote yield and sustainability^2^. It is projected that a 1% increase in the NUE of crops could save over a billion dollars annually, which could be a considerable economic benefit^3^.

Despite the advances in high throughput phenotyping and genetic resources in major crops (rice, maize, wheat, soybean) to evaluate traits associated with N utilization and productivity, such knowledge of N-induced changes in the biomass in leafy vegetables is still emerging. Commercial spinach producers use high levels of N fertilizers to produce higher biomass and better quality like many leafy greens. Spinach has poor NUE^4–6^ due to its shallow root system^7^ and short production cycle. N plays a critical role in root and shoots communications to maximize plant productivity and agronomic applications^8^. Enhancing the NUE in spinach by exploiting genetic diversity in the leaf traits has been proposed^9,10^. We have previously shown that using a uniform soil-less matrix allowed us to use the supervised machine learning algorithm to predict the importance of root traits in spinach^11^. Biomass allocation among above (leaves) and below-ground (roots) organs is influenced by N uptake and distribution, which have crucial impacts on plant growth and development. N-induced changes in the shoot and root biomass have practical value as selection tools in vegetable breeding programs.

Genome-wide association studies (GWAS) have proven to be a powerful tool for characterizing the genetic constitution and identifying potential candidate genes associated with traits of interest. With the advent of genome editing techniques, it is now possible to manipulate the genetic makeup and facilitate speed breeding to enhance NUE by employing the genes identified by GWAS, selective sweeps, or other computational methods.

In this study, we used GWAS to analyze percent changes in the shoot and root biomass in spinach at two N regimes to (1) depict the phenotypic variation in the biomass of shoot and root under low and high N levels, (2) categorize SNP markers associated with these changes, and (3) identify candidate genes influenced by N perturbation. Identifying QTLs for N-induced responses to N uptake offers a promising way in leafy greens to optimize N fertilizer requirements while maintaining a balance between assimilated N and vegetative growth needed for yield stability.

## Methods

### Plant material and phenotyping

Seeds of 201 spinach (*S. oleracea*) accessions obtained from the USDA-National Plant Germplasm System (NPGS) (https://npgsweb.ars-grin.gov/) at the USDA-ARS North Central Regional Plant Introduction Station, Iowa State University, Ames, Iowa, USA were used for the study. The plants were grown in a growth chamber at the Texas A&M AgriLife Research and Extension Center, Uvalde, Texas, in 2020, under controlled conditions of 12/12 h (light/dark), 22 °C, and 75% relative humidity. The seeds of each accession were sown in triplicate in turface (Turface Athletics MVP, PROFILE Products LLC, Buffalo Grove, Illinois, USA) in small pots (10.2 cm × 10.2 cm and 8.9 cm deep) following a randomized complete block design. Additional details of the experimental set-up are detailed previously^11^. Plants were fertilized with two concentrations of N, low N (LN; 50 ppm), and high N (HN; 200 ppm) using Peters® professional ready mix (5-11-26, hydroponics special water-soluble fertilizer, Everris NA Inc., Ohio, U.S.A.) after seedling emergence. The plants were harvested after 41 days for the above-ground shoot and root biomass by carefully separating roots from the Turface. The fresh shoot and root weights were measured and expressed as % change (100 × LN/HN) for each accession. The accessions used for the study were distributed across 28 countries (Table  S1, Fig S1), with a majority (over 80%) from ten countries: Turkey (n = 65), Afghanistan (n = 17), North Macedonia (n = 17), China (n = 15), Iran (n = 11), India (n = 11), United States (n = 7 plus 8 developed). The analysis of variance (ANOVA) with the general linear models (GLM) for the phenotypic data of the 201 spinach accessions was performed in JMP Genomics 9 (SAS Institute, Cary, NC). The student T-test was used to perform multiple comparisons of accessions at α = 0.05. The average % changes in shoot and root biomass for each accession were used for GWAS. The plant collection and use was in accordance with all the relevant guidelines.

### Genotyping and SNP selection

DNA extractions and sequencing details are provided in our previous publications^12–14^. The spinach genome of Sp75 at SpinachBase (http://www.spinachbase.org) was used as a reference for mapping the Illumina short reads of 201 spinach accessions using Burrows-Wheeler aligner software (BWA, 0.7.8 http://bio-bwa.sourceforge.net/)^15^. SAMtools (0.1.19; http://samtools.sourceforge.net) was used for sorting the indexed binary alignment map files. The program Picard (v.1.111; (http://picard.sourceforge.net)) was used to remove duplicate reads and merge the bam files from the same sample. Genome Analysis Toolkit software (GATK, version v.3.5; http://www.broadinstitute.org/gatk/)^16^ was used to detect and filter SNPs and InDels, while ANNOVAR^17^ was used to annotate all sequence variants. Around 2.357 million SNPs were detected in the 201 spinach genotypes. After filtering and keeping the SNPs with minor allele frequency (MAF) > 2% and missing allele < 15%, heterogeneous rate < 35%, 60,940 SNPs distributed on six chromosomes (Chr) were used in this study. The SNPs distribution across chromosomes was as follows; 6,788 SNPs on Chr 1; 6,134 SNPs on Chr2; 17,931 SNPs on Chr 3; 12,563 SNPs on Chr 4; 11,830 SNPs on Chr 5; and 5,694 SNPs on Chr 6 (Fig. S2, FigShare Table S2). The SNP datasets for 201 spinach accessions phenotyped for the shoot and root weights (biomass) were used in this study.

### Principal component analysis and genetic diversity

In this study, the 60,940 SNPs (FigShare Table S2) were used for principal component analysis (PCA) and genetic diversity analysis by GAPIT 3 (Genomic Association and Prediction Integrated Tool version 3)^18,19^. The neighbor-joining (NJ) method built-in GAPIT 3 was used to draw phylogenetic trees.

### Association analysis and candidate gene identification

We used the 60,940 SNPs to perform association analysis in TASSEL 5 (Bradbury et al.^20^; http://www.maizegenetics.net/tassel) MLM (PCA + K). PCA matrix was estimated by the PCA tool in TASSEL 5, where covariance (alterative = correction) and the number of components = 2 were set. Kinship (K) was estimated by the tool Kinship with Scald_IBS method in TASSEL 5. The GWAS was also performed by BLINK (bayesian-information and linkage-disequilibrium iteratively nested keyway) model in GAPIT 3 (Genomic Association and Prediction Integrated Tool version 3) (https://zzlab.net/GAPIT/index.html; https://github.com/jiabowang/GAPIT3) ^21,22^ by setting PCA = 2 using the 60,940 SNPs. Significant threshold of associations was determined using Bonferroni correction of P-value at an α = 0.05 (0.05/SNP number) (López-Hernández and Cortes, 2019). In this study, for the panel of all 201 accessions, the significant –log(P-value) threshold value was 6.09 based on the 60,940 SNPs. Candidate genes were searched within 100 kb on either side of significant SNPs. Candidate genes were retrieved from the spinach Sp75 genome reference annotation at the SpinachBase site (http://www.spinachbase.org/).

## Results

### Phenotypic analysis

**Figure 1:**
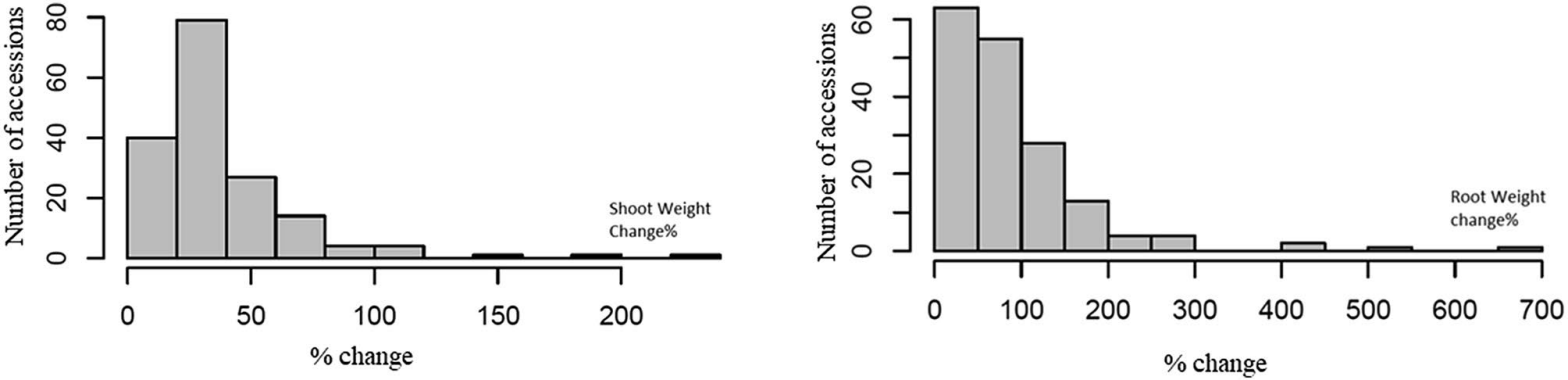
The distribution of percent changes in the shoot (**A**) and root (**B**) weight (biomass) across spinach accessions. Forty-one-day plants were harvested for the shoot and root biomass and expressed as % change (100 × LN/HN; shown on the horizontal axis) for 201 accessions (vertical axis).

A total of 201 spinach accessions were used in this study. The percent changes in the shoot and root biomass are presented in Fig. 1 (Table S1). Although the data showed an extensive range in percent change in biomass for both the tissue types across spinach accessions induced due to N, the distribution was skewed, suggesting a limited number of accessions showed increased shoot or root biomass. Pearson’s correlation among percent changes in shoot and biomass was highly correlated (r = 0.62). Across all the accessions, PI648964, PI249920, PI169669, PI103063, and PI179592 showed the highest percent increase in shoot biomass, while PI181808, PI648964, PI226671, PI212120, and PI103063 showed the highest increase in percent root biomass. PI648964 and PI103063 showed higher percent changes in both tissue types.

### PCA and phylogenetic analysis

**Figure 2:**
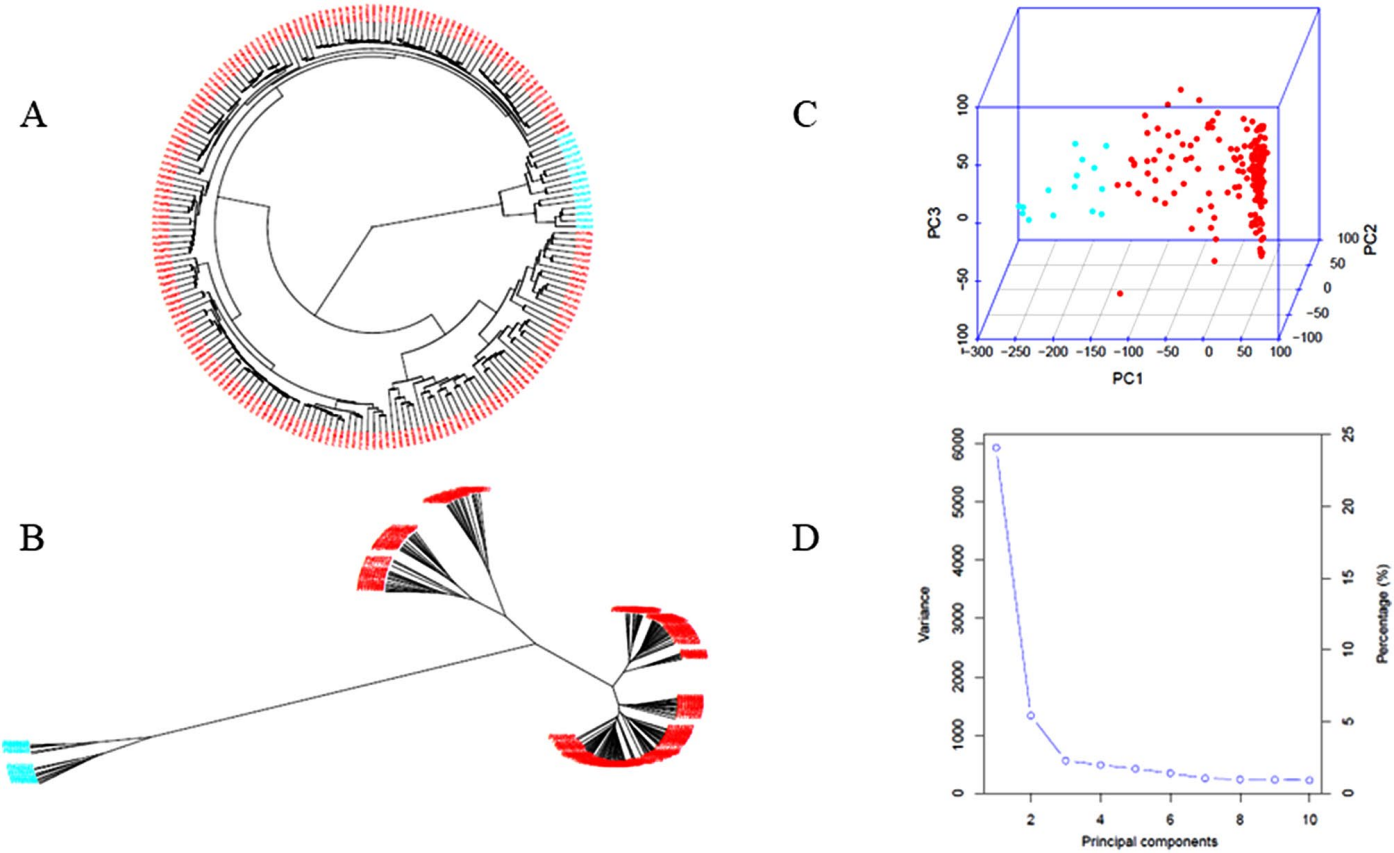
Population genetic diversity analysis of 201 USDA spinach accessions. Phylogenetic trees drawn by neighbor-joining (NJ) method showing in (fan) (**A**). unrooted (**B**), and 3D graphical plot of the principal component analysis (PCA) in two sub-population (**C**) and PCA eigenvalue plot drawn (**D**) drawn by GAPIT 3. A readable phylogenetic tree for each accession is shown in Supplementary Fig. S2.

Two sub-populations (clusters) dividing in the GWAS panel of the 201 accessions were identified based on PCA using the 60,940 SNPs distributed 6 chromosomes and phylogenetic analysis by neighbor-joining (NJ) method drawn by GAPIT 3 (Fig. 2, a detailed phylogenetic tree in Fig S3). Based on the 2-cluster, Q1 and Q2 consisted of 186 (92.5%) and 15 (7.5%) accessions. Most of the accessions from different countries were grouped into Q1, while selected accessions from India and China were grouped into Q2. The genetic relationship among the accessions was visualized in the heatmap of the distance matrix (Fig. S4).

## Association analysis

We performed BLINK analyses in GAPIT 3 and MLM (PCA + K) analyses in TASSEL 5, by setting PCA = 2 for the panel of 201 spinach accessions using 60,940 distributed across six chromosomes (Fig. S2) filtered SNPs. Both models selected SNPs with LOD > 6.09 as associated SNP markers. Combined output for both models for % root and shoot weights with significant SNP markers are shown in Table 1.

**Table 1:** SNP markers associated with % changes in shoot and root weights in spinach accessions, based on two models, MLM in Tassel 5 and BLINK in GAPIT 3.

Trait	Marker	Chr	Position	-Log(*P*-value) in Tassel MLM	-Log(*P*-value) in GAPIT3 BLINK	MAF%	R^2^ (%)
% Shoot wt	chr2_18480277	2	18,480,277	7.25	1.51	21.6	22.1
% Shoot wt	chr4_47598760	4	47,598,760	6.74	5.74	20.1	21.1
% Root wt	chr4_28292655	4	28,292,655	6.15	3.85	19.6	19
% Root wt	chr6_1531056	6	1,531,056	6.28	5.97	20.2	20.1
% Root wt	chr6_37966006	6	37,966,006	7.26	6.61	21.6	23.1

**Figure 3:**
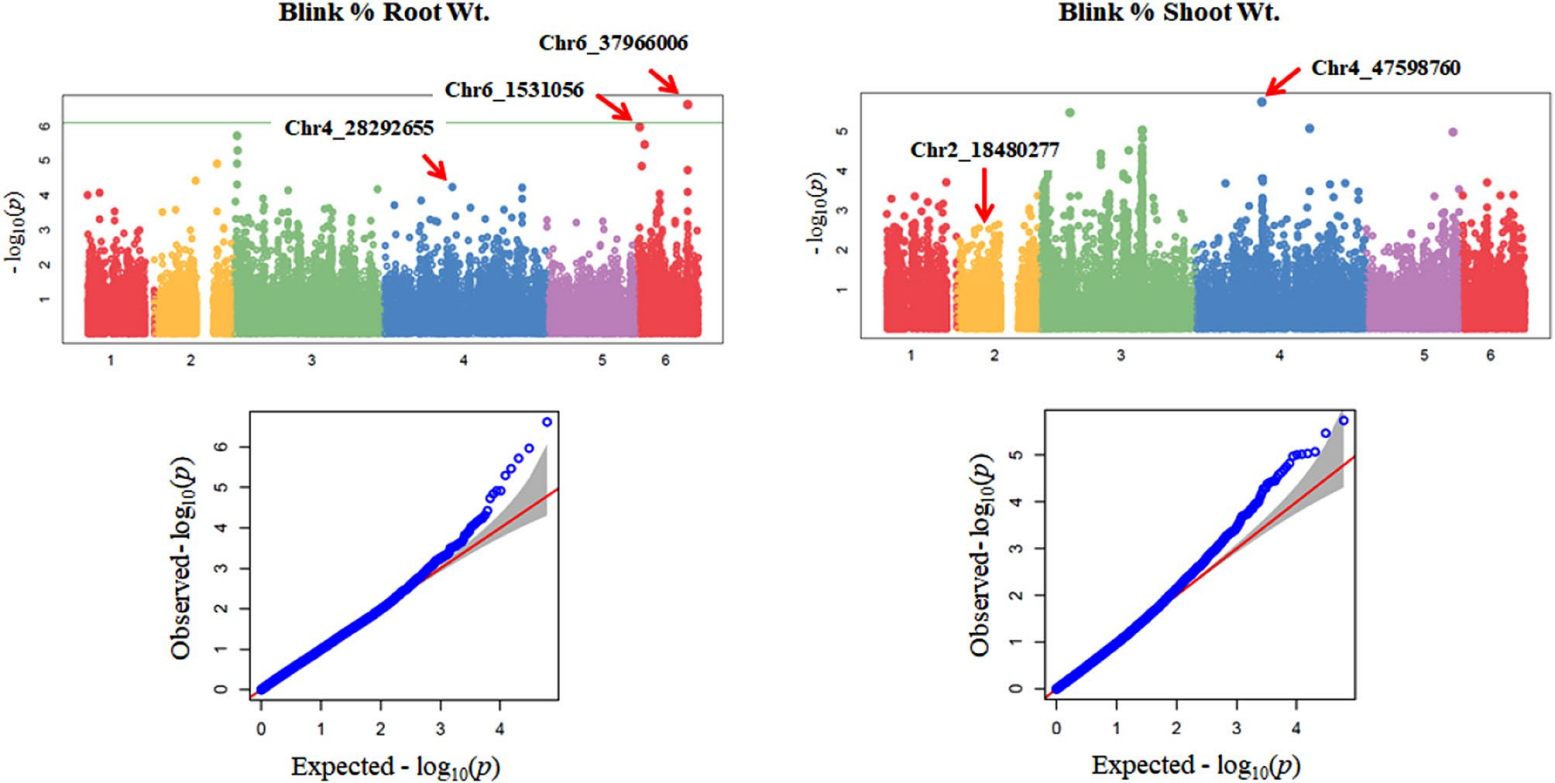
Distributions of Manhattan and QQ-plots of GWAS based on BLINK for % root and shoot weights, where x-axis represents the spinach chromosomes and y-axis represents LOD [–log(*P*-value)] value of each SNP in Manhattan plot, and x-axis represents the Expected LOD [–log(*P*-value] and y-axis represents Observed LOD [–log(*P*-value)] value of each SNP in QQ-plot.

**Figure 4:**
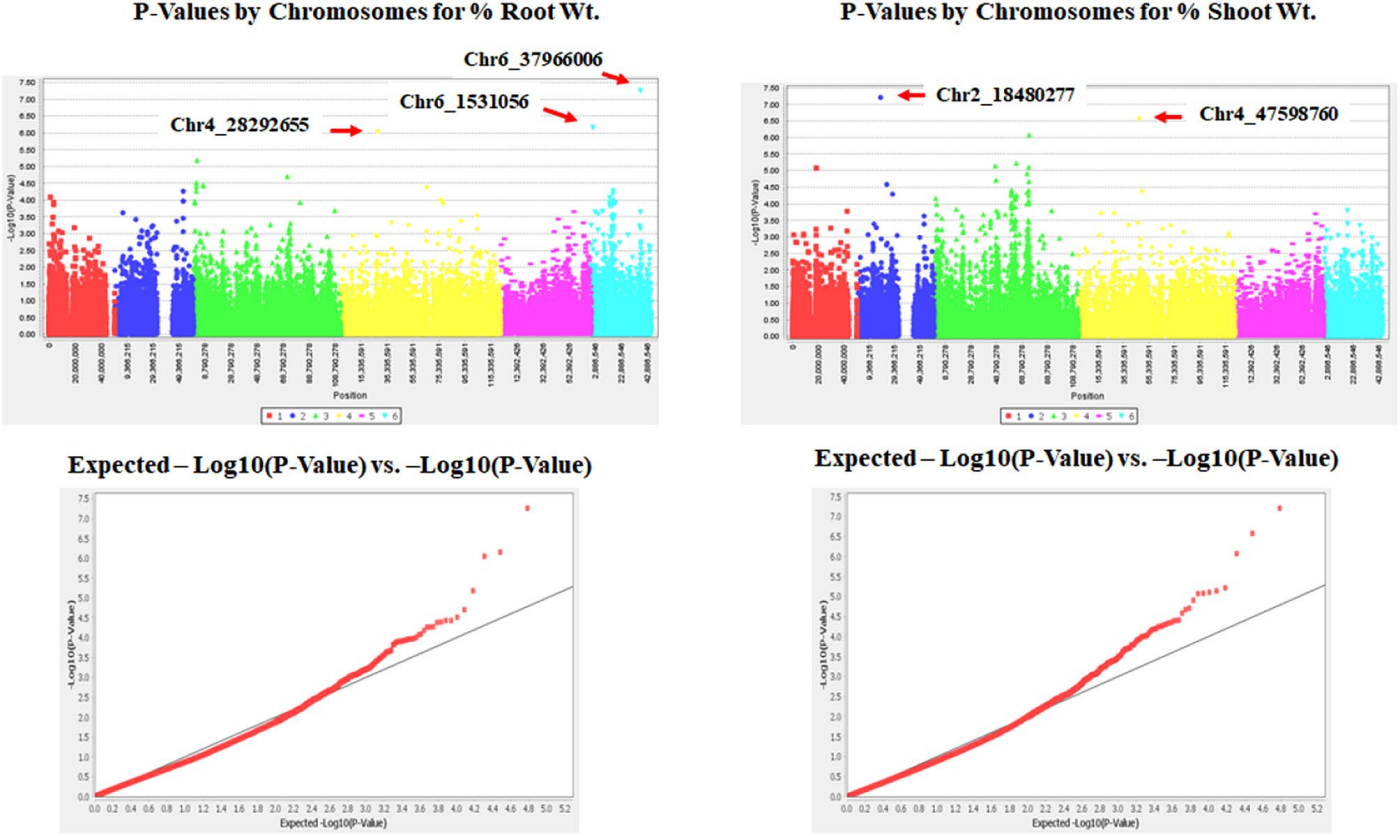
Distributions of Manhattan and QQ-plots of GWAS based on MLM for % root and shoot weights, where the x-axis represents the spinach chromosomes and the y-axis represents LOD [–log(P-value)] value of each SNP in Manhattan plot, and x-axis represents the Expected LOD [–log(*P*-value)] (shown in red color), and the y-axis represents Observed LOD [–log(*P*-value)] (shown as black line) value of each SNP in QQ-plot.

Based on BLINK MLM model, the distributions of the QQ plots between the observed vs. expected LOD [−log10(p)] showed a moderate divergence from the expected distribution for both % change in root and shoot weights (Fig. 3), suggesting the presence of SNPs associated with both traits in the association panel. The Manhattan plot showed SNPs with LOD value greater than 6.09 (significant threshold) only for % root weight. Based on MLM (PCA + K) model in TASSEL 5, QQ plots showed a divergence distribution for both % change in root and shoot weights, and the Manhattan plot showed SNPs with LOD values greater than 6.09 for both traits (Fig. 4), suggesting the presence of SNPs associated with both traits in the association panel. Combined with two model analyses, three SNP markers, chr4_28292655, chr6_1531056, and chr6_37966006 were significantly associated with %change in root weight and two SNP markers, chr2_18480277 and chr4_47598760 were significantly associated with %change shoot weight. The SNP marker chr6_37966006, located at 28,292,655 bp on chromosome 6, had a significantly high LOD value at MLM in Tassel and Blink in GAPIT for % root weight indicating this marker was associated with % root weight increase due to N across both the tested models. While markers chr6_1531056 at 1,531,056 bp on chr6 and Chr4_28292655 at 28,292,655 bp on Chr 4 had higher LOD than the threshold value for MLM in Tassel but not in the case of Blink model, indicating less stability across models. Similarly, both the markers (chr2_18480277 and chr4_47598760) for % shoot weight showed higher LOD values than the threshold only for the MLM tassel model.

## Candidate Genes for percent shoot and root increases

Significant SNPs were aligned against the spinach Sp75 reference genome, and regions within 100 kb were searched for candidate genes. A total of 13 and five unique genes were identified for % root and shoot weight, respectively, within these regions (Table 2), most of which had functional annotation and were associated with regulatory developmental or physiological processes. For % root weight, the O-fucosyltransferase family protein-coding gene (Spo07469) was located on the marker chr6_37966006. In comparison, six (Spo07470, Spo07468, Spo07458, Spo07459, Spo07460, Spo07474) genes were found within a 10–100 kb distance of the marker chr6_37966006 and three (Spo26174, Spo26178, Spo26172) genes within 5-100 kb distance of the marker chr6 1,531,056 on chromosome 6. Three genes were located close to the marker chr4_28292655 for % root weight. For % shoot weight, we discovered two (Spo22959 and Spo22961) and three (Spo20482, Spo20477, and Spo20481) candidate genes near markers on chromosomes 2 and 4, respectively.

**Table 2:** List of candidate genes located at associated SNP regions for % change in root and shoot weights.

Trait	SNP	Chr	Pos	Distance		Gene	Start	End	Description	Distance from the SNP and gene
% Root Wt	chr6_37966006	6	37,966,006	1897	− 4579	Spo07469	37,964,109	37,970,585	O-fucosyltransferase family protein	On gene
				− 5172	− 11,479	Spo07470	37,971,178	37,977,485	Unknown protein	Within 10 kb
				10,483	6592	Spo07468	37,955,523	37,959,414	DUF538 family protein	Within 10 kb
				20,389	13,939	Spo07458	37,945,617	37,952,067	Transcription factor bHLH14	Within 20 kb
				− 12,392	− 24,135	Spo07459	37,978,398	37,990,141	Mechanosensitive channel of small conductance-like 6, putative	Within 20 kb
				− 47,093	− 51,458	Spo07460	38,013,099	38,017,464	Abscisic acid receptor PYL2 (PYR1-like protein 2) (Regulatory components of ABA receptor 14)	Within 50 kb
				− 98,341	− 108,490	Spo07474	38,064,347	38,074,496	Breast cancer 2 susceptibility protein	Within 100 kb
	chr6_1531056	6	1,531,056	− 4353	− 17,109	Spo26174	1,535,409	1,548,165	Unknown protein	Within 5 kb
				46,378	39,422	Spo26178	1,484,678	1,491,634	TRAF-like family protein, putative	Within 50 kb
				− 75,679	− 91,041	Spo26172	1,606,735	1,622,097	Splicing factor U2af large subunit B	Within 100 kb
	chr4_28292655	4	28,292,655	− 27,236	− 32,810	Spo23961	28,319,891	28,325,465	Unknown protein	Within 30 kb
				− 41,374	− 69,747	Spo23960	28,334,029	28,362,402	Imidazole glycerol phosphate synthase subunit HisF	Within 50 kb
				− 80,274	− 96,844	Spo23948	28,372,929	28,389,499	tRNA pseudouridine synthase (5.4.99.-)	Within 100 kb
% Shoot Wt	chr2_18480277	2	18,480,277	28,405	7215	Spo22959	18,451,872	18,473,062	Protein kinase	Within 10 kb
				− 98,011	− 107,689	Spo22961	18,578,288	18,587,966	Transducin/WD-40 repeat protein family-like protein	Within 100 kb
	chr4_47598760	4	47,598,760	30,976	12,183	Spo20482	47,567,784	47,586,577	Unknown protein	Within 20 kb
				22,833	20,436	Spo20477	47,575,927	47,578,324	HAT family dimerisation domain containing protein	Within 30 kb
				36,165	31,586	Spo20481	47,562,595	47,567,174	RING/U-box superfamily protein, putative	Within 50 kb

## Discussion

Applying N-rich fertilizers for more food production is continually rising with the growing demand. Vegetables like spinach are often over-fertilized for higher production, enhancing appearance traits such as leaf greenness, brightness, or luster. However, vegetable growers are under increasing regulatory pressure to improve nutrient management and reduce NO_3_^−^ losses to ground and surface waters. Spinach is often regarded as one of the highest NO_3_^−^ accumulator vegetables due to its relatively poor nitrate reduction efficiency^4,23,24^. Although spinach is a rich source of specific health-promoting nutrients, it accumulates high amounts of anti-nutrient oxalates^25^. Studies have shown increased oxalate accumulation^26–28^, and reduced quantities of beneficial nutrients like ascorbic acid and flavonoids^24^ with increasing N amounts in spinach leaves. Spinach breeders thus have a significant challenge to mitigate these adverse effects by developing varieties that can utilize lower N input without compromising productivity. About 60% of N in spinach production is lost through leaching primarily due to its shallow root system^7^, inability to access NO_3_^-^ below the root zone^29^, and short production cycle. Although several studies have confirmed the genotypic differences in N accumulation in spinach^4,30^, developing varieties responding to increased N by producing higher shoot biomass and expansive root systems would benefit high-density commercial production. Although higher yield with high N is a significant incentive for growers, given the high production cost and environmental damage, such varieties that can efficiently partition the N in leaf and root biomass will be rewarding. As the success of a breeding program for improving specific traits depends on existing genetic variability in the base population, it is necessary to define the genetic variation in the available germplasm for NUE traits for introgression into varieties. This study identified several accessions that showed the highest percent increase in shoot and root biomass which can be used as inbreds in breeding programs to enhance the NUE of cultivated varieties.

In this study, GWAS was performed using the MLM model in both GAPIT3 and Tassel 5 programs to identify SNP markers associated with the percent changes in the shoot and root biomass in response to N. Three SNP markers, chr4_28292655, chr6_1531056, and chr6_37966006 on chr4 and chr6 were significantly associated with % change in root weight, and two SNP markers, chr2_18480277 and chr4_47598760 on chr2 and chr4 were significantly associated with % change shoot weight (Table 1). Based on the MLM model in both GAPIT3 and Tassel 5, the SNP marker, chr6_37966006 at 37,966,006 bp on chromosome 6 was the best marker identified in this study. It had a high LOD value with 6.61 or 7.26, respectively, from GAPIT3 and Tassel 5 for the % change in root weight response to N. The SNP explained a high phenotypic variation with 23.1% R^2^ value (Table 1), suggesting that there is a QTL in the region of the chromosome 6 for NUE under root response. On the other side, the SNP marker, chr4_47598760 at 47,598,760 bp was also a good marker for the % change in shoot weight response to N with a high LOD value of 5.74 or 6.74, respectively, as per GAPIT3 and Tassel 5, and explained a high variation (21.1% R2 value) (Table 1), suggesting that there is a QTL in the region of the chromosome 4 for NUE in shoot response. A previous study^10^ also reported two QTLs for shoot dry weight, one at low N and one at high N at P01 linkage group (LG) with a peak at 3.8 cM, explaining 15.1 % phenotypic variation for low N and 13.9 % for high N; two QTLs at P01 and P05 LGs for shoot fresh-weight under low N, 29.8 % of the variation; and a QTL for dry matter percentage on P05 under low N. Whether the QTLs identified in this study are the same as those reported earlier would need experimental validation.

This study identified the O-fucosyltransferase family protein as one candidate gene enhancing root weight. The O-fucosyltransferase SPINDLY (*SPY*) is one of the enzymes involved in O-glycosylation in higher plants that play a role in plant growth and development^31^. A SPY-dependent protein O-fucosylation of DELLAs, transcription regulators of gibberellin (GA) signaling, is critical in regulating plant development^32^. A molecular approach that evaluated the role of GA metabolism and signaling in nitrate-regulated growth processes in Arabidopsis and wheat showed that nitrate increased bioactive GA levels and the degradation of DELLAs, thereby activating cell proliferation and root and shoot growth^33^. The role of O-fucosylation in establishing root hair cell patterning has been recently validated in Arabidopsis by showing a defective root hair patterning in O-fucosyltransferase SPINDLY (*SPY*) loss-of-function mutant^34^. Root hairs play an essential role in water and nutrient uptake by impacting root surface area. The distribution pattern of root hairs is regulated by hormones and external stimuli like nutrient availability. Functional validation of the putative gene Spo07469 identified by the SNP marker chr6_37966006 in enhancing root biomass would help understand its significance in N acquisition and serving as a serve marker for introgression breeding in spinach. The basic helix-loop-helix (bHLH) transcription factors (TF) represents one of the most prominent TF families in plants involved in a broad range of biological functions, including the root and shoot cell fate determination^35^. Many bHLH TF family members develop root hair cells critical to nutrient and water absorption and interact with soil microbiomes^35–37^. Specifically, bHLH14, which was identified in our study (Spo07458), acted as a transcription repressor to negatively regulate JA responses in Arabidopsis quadruple mutant with defective bHLH genes showing severe severity sensitivity to JA-inhibited root growth^38^. Mechanosensitive channels of small conductance (MscS) are pore-forming transmembrane proteins involved in the movement of ions across the plasma membrane across the electrochemical gradient. Characterization of MscS-Like (MSL) channels in Arabidopsis suggested their mechanosensitive activities in the plasma membrane of root cells^39^. Upon exposure to various stimuli, these proteins have been proposed to increase cytosolic Ca^2+^ concentration or activate other Ca^2+^ channels^40^. It is plausible that the gene Spo07459 identified in this study may play a similar role in root cells to regulate ion exchange in response to N availability.

Another candidate gene, Spo07468 encoding DUF538 family protein, was identified for % root weight. These proteins have been characterized in stress-challenged plants, including those grown under nutrient deficiency and drought stresses^41,42^. It is also proposed that DUF538 like proteins may help the catabolism of pectin in the cytosol to recycle carbon in stress-challenged plants^43^. Our study also identified a protein kinase (Spo22959) gene for % shoot weight increase in response to N, showing similarity (73%) to Arabidopsis (At2g40730) N-terminal protein kinase like-domain protein involved in cytoplasmic tRNA export^44^.

We found 18 candidate genes close to significant markers in the spinach panel within a 100-kb region window in either direction of a significant SNP. The most significant SNP marker, chr6_37966006 was located on O-fucosyltransferase (Spo07469) gene. This gene is likely to play a critical role in regulating root growth, and development in spinach in response to N. Additional studies would facilitate validation of identified genes and their integration into the spinach breeding programs to enhance NUE. The application of nitrogen-rich fertilizers for improved crop production is continually rising. Developing spinach cultivars to increase production proportionate to the applied nitrogen would allow efficient nitrogen management. We observed a large phenotypic variation as percent changes in the shoot and root biomass in response to N among a spinach panel of 201 accessions and identified significant associations between SNPs markers and increases in root and shoot biomass in the present study. These markers will help improve the efficiency of spinach breeding programs and represent necessary steps toward the selections for NUE parental lines for developing Kompetitive Allele-Specific PCR (KASP) markers to be used in marker-assisted selection (MAS) and future introgression breeding.

## Supplementary Information

Supplementary Information 1.

Supplementary Information 2.

Supplementary Information 3.

Supplementary Information 4.

Supplementary Information 5.

Supplementary Information 6.

## Data Availability

All genomic sequencing raw data generated and used for the spinach accessions in this study have been deposited at the National Center for Biotechnology Information (NCBI) Sequence Read Archive (SRA) with BioProject: PRJNA860974. All other data generated or analyzed during this study are included in this published article (and Supplementary Information files). FigShare_Table_S2 https://doi.org/10.6084/m9.figshare.19684041.v1.
