# Supplementary figures and images for: Genetic dissection of nitrogen induced changes in the shoot and root biomass of spinach
Source: Sci Rep. 2022 Aug 12;12:13751. doi: 10.1038/s41598-022-18134-7 (PMC9374745; doi:10.1038/s41598-022-18134-7)

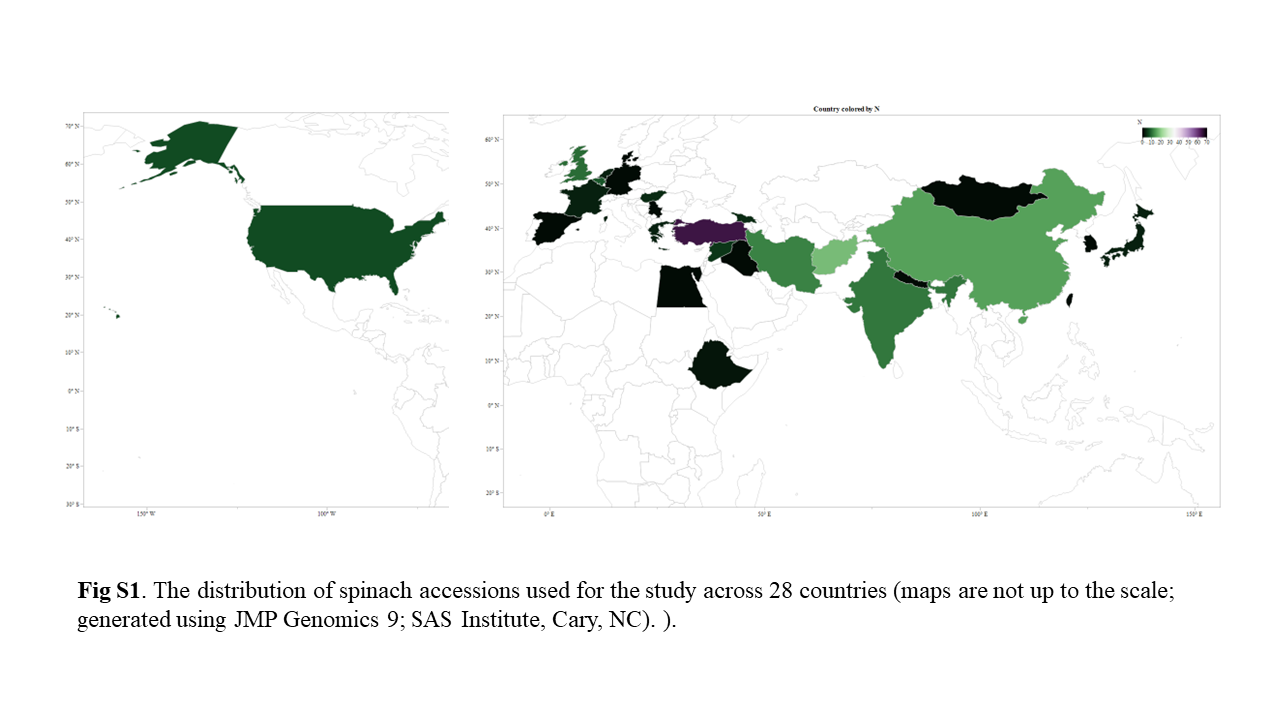

Supplement: Supplementary file 1 — Supplementary Information 1. [file 41598_2022_18134_MOESM1_ESM.png]

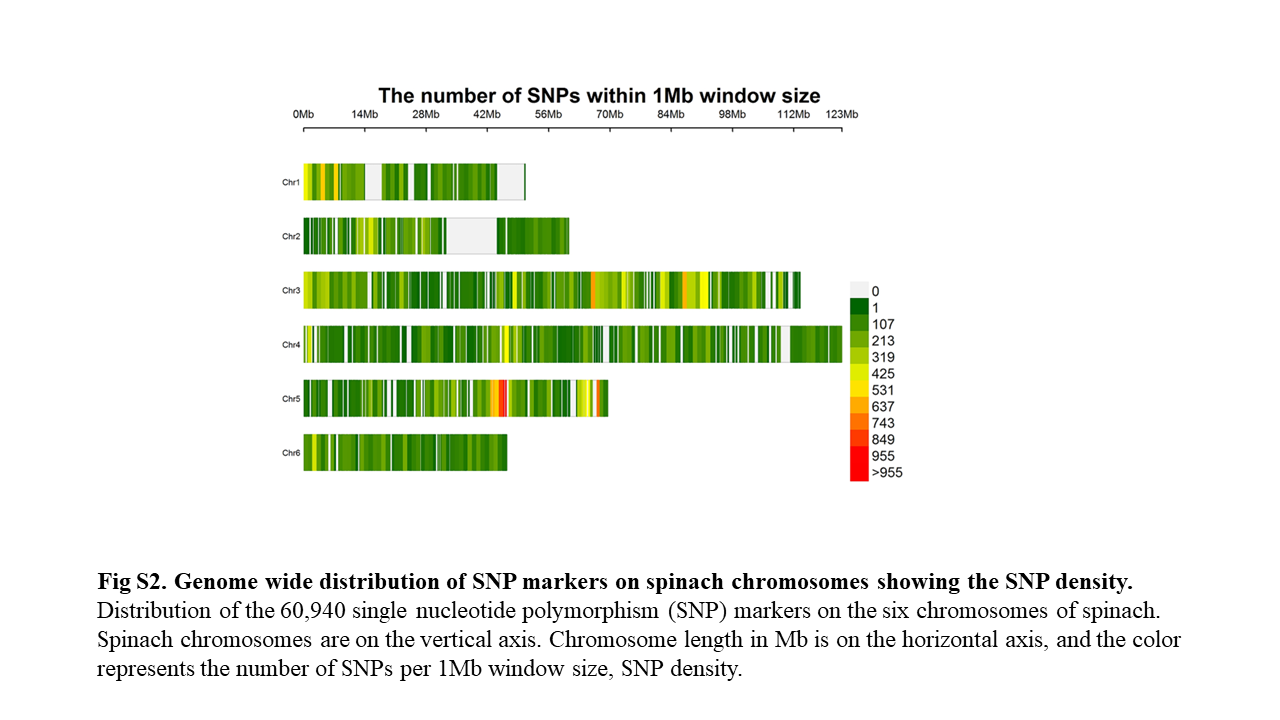

Supplement: Supplementary file 2 — Supplementary Information 2. [file 41598_2022_18134_MOESM2_ESM.png]

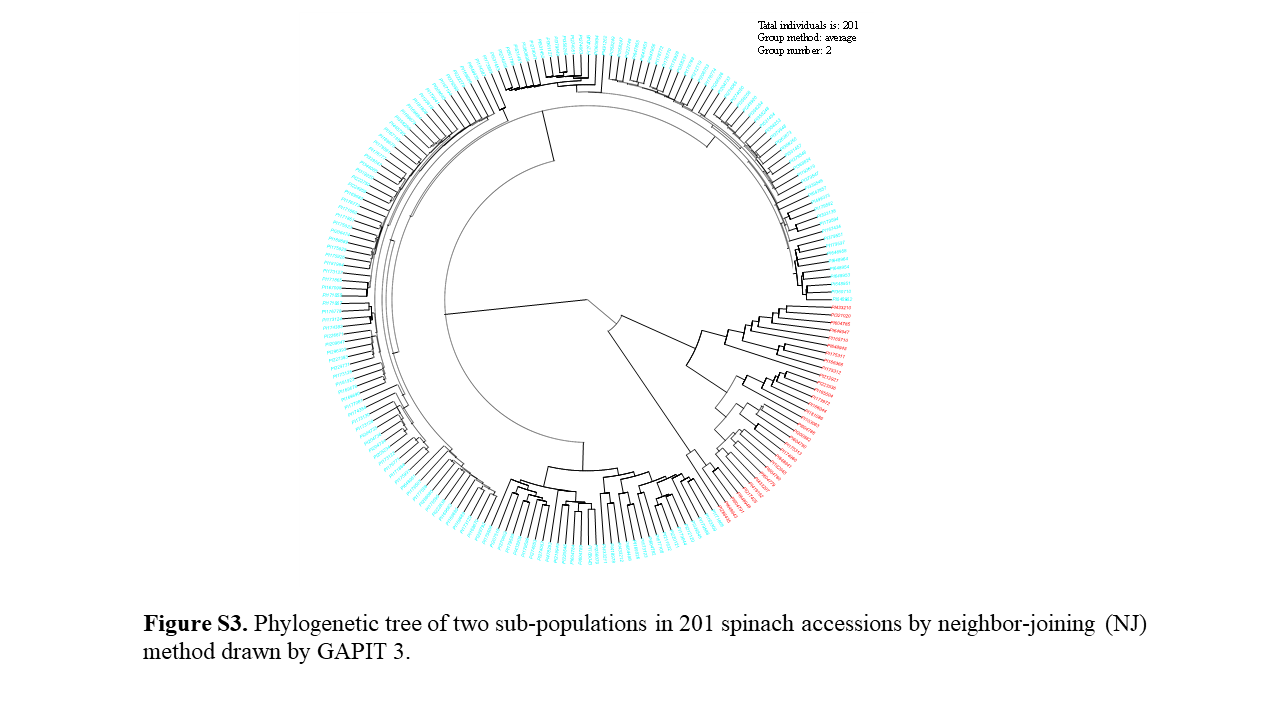

Supplement: Supplementary file 3 — Supplementary Information 3. [file 41598_2022_18134_MOESM3_ESM.png]

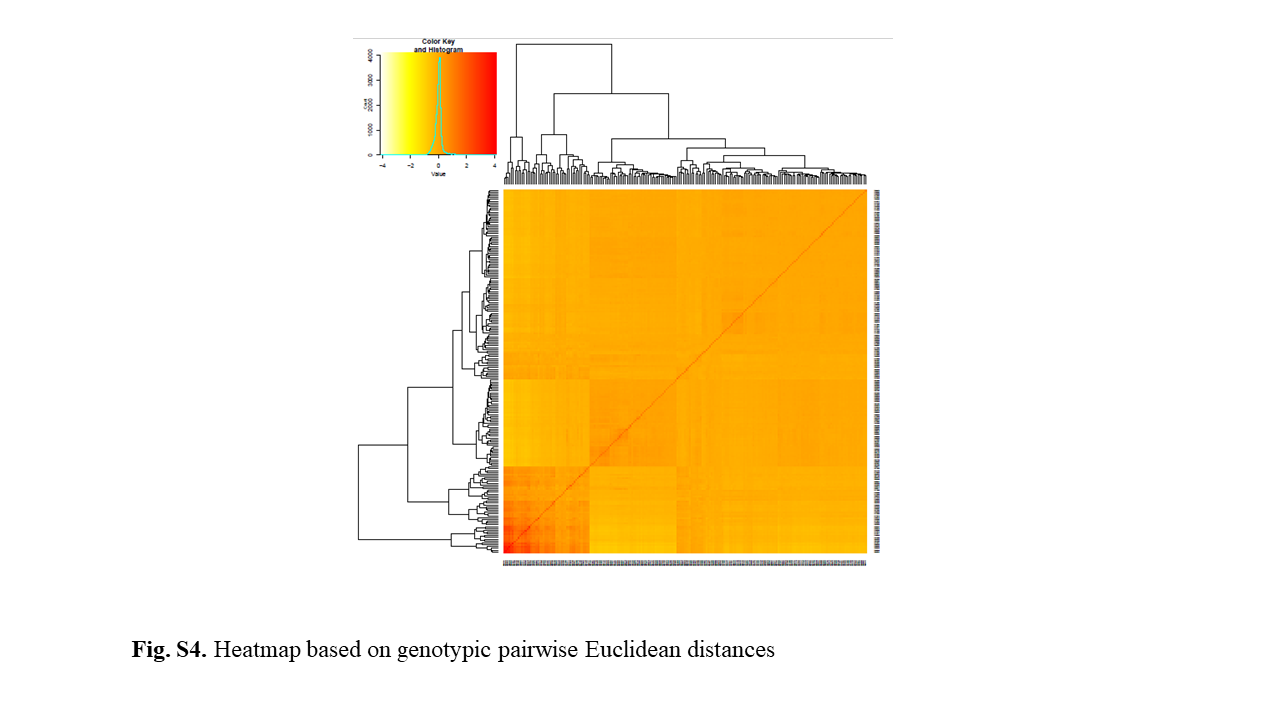

Supplement: Supplementary file 4 — Supplementary Information 4. [file 41598_2022_18134_MOESM4_ESM.png]
